# A rare presentation of dyschromatosis symmetrica hereditaria in a Canadian context: A case report

**DOI:** 10.1177/2050313X251358966

**Published:** 2025-07-31

**Authors:** Sonia Czyz, Fatemeh Jafarian, Karen Holfeld

**Affiliations:** 1 Department of Medicine, University of Calgary, Calgary, AB, Canada; 2 Division of Dermatology, Department of Medicine, University of Calgary, Calgary, AB, Canada; 3 Division of Dermatology, College of Medicine, University of Saskatchewan, Regina, SK, Canada

**Keywords:** pigmentary disorder, genodermatoses, pathology, pediatrics

## Abstract

Inherited reticulate pigmentary disorders (IRPD) are a group of rare dermatologic conditions characterized by distinct reticulate patterns of hyperpigmentation and/or hypopigmentation of the skin. These conditions are considered genodermatoses caused by genetic mutations that are often inherited in affected families. IRPDs exhibit considerable phenotypic variability, ranging from having minimal or no systemic involvement to profound associations with neurocognitive, immunologic, and other organ abnormalities. Here, we discuss a rare case of dyschromatosis symmetrica hereditaria in an otherwise healthy 6-year-old boy. In this report, we aim to present the clinical manifestations of one subtype of reticulate pigmentary genodermatosis that, although uncommon in regions like Canada, immigration from other parts of the world, like Asia, highlights the need for awareness among Canadian physicians.

## Introduction

Dyschromatosis symmetrica hereditaria (DSH) was first described in the early 1900s in Japan, initially known as reticulated acropigmentation of Dohi.^1^ It is a rare pigmentary genodermatosis of autosomal dominant inheritance with high penetrance.^2,3^ The condition is characterized by diffuse, symmetrically distributed hyperpigmented and hypopigmented macules on the dorsal surfaces of distal extremities, often accompanied by freckle-like macules on the face. The pigmentary changes generally first appear in infancy or early childhood.^3,4^ Once the lesions establish, they persist throughout life without changes in color or distribution.

DSH has mainly been described in Japanese and Chinese patients; however, cases have also been reported in patients of Indian, Korean, European, Nigerian, Turkey, and Hispanic origin.^3,5–7^ Although pathogenesis remains largely unclear, mutations of the double-stranded RNA-specific adenosine deaminase acting on the ribonucleic acid 1 (ADAR1) gene located on chromosome 1 have been reported in affected individuals.^8–11^ ADAR1 is widely expressed in the skin and postulated to contribute to pigmentary alteration at the molecular level, impacting melanocyte differentiation, distribution, and apoptosis.^12,13^ Generally, DSH is considered an isolated cutaneous disorder with minimal systemic associations, particularly when compared to other IRPDs (Table 1).^14^

**Table 1.** Inherited reticulate pigmentary disorders are characterized by distinct patterns of pigmentation, histopathological findings, genetic mutations, and systemic involvement.

IRPD subtype	Pigmentation presentation	Histopathology	Gene	Inheritance	Systemic involvement
Dyschromatosis symmetrica hereditaria	Hyperpigmented and hypopigmented macules on dorsal distal extremities, ± pigmented freckle-like lesions on the face.	Hyperpigmented macules show increased epidermal basal layer melanin. Hypopigmented macules show reduced and apoptotic melanocytes.	ADAR1	AD	Usually none, may be associated with neurologic or cardiac changes.
Dyschromatosis universalis hereditaria	Hyperpigmented and hypopigmented macules are distributed generally over the body.	Hyperpigmented macules show increased melanin content in basal layer and melanin incontinence in papillary dermis. Hypopigmented macules show decreased melanin in basal layer. Melanocyte number often normal.	ABCB6, SASH1	AD or AR	Dystrophic nails, and sometimes abnormalities of hair and oral mucosa.
Reticulate acropigmentation of kitamura	Well-defined, ± depressed, hyperpigmented macules on the hands and feet, often with palmar pits.	Epidermal atrophy, elongated rete ridges, increased melanocytes, and hypermelanosis.	ADAM10	AD	None.
Dowling-degos disease	Reticulate hyperpigmentation in flexural areas (axilla, infra-mammary folds, inguinal folds, neck).	Thin epidermis with a typical ‘antler-like’ appearance of elongated, slender, branched rete ridges	KRT5, POFUT1, POGLUT1, PSENEN and possibly GLMN	AD	None.
X-linked reticulate pigmentary disorder	Females: Hyperpigmented patches distributed along the lines of Blaschko. Males: Generalized reticulate hyperpigmentation and hypopigmentation.	Mild hyperkeratosis, acanthosis, hyperpigmentation of the basal layer, and melanin incontinence in the upper dermis.	POLA1	X-linked	Females: None. Males: Immunodeficiency, recurrent infections, ichthyosis, bronchiectasis, eosinophilic gastroduodenitis, photophobia.

ABCB6: ATP-binding cassette subfamily B member 6; AD: Autosomal dominant; ADAM10: A Disintegrin and Metalloprotease 10; ADAR1: Adenosine deaminase acting on RNA 1; GLMN: Glomulin KTR5: Keratin 5; AR: Autosomal recessive; POLA1: DNA Polymerase Alpha 1, Catalytic Subunit; POFUT1: Protein O-Fucosyltransferase 1; POGLUT1: Protein O-Glucosyltransferase 1; PSENEN: Presenilin Enhancer, Gamma-Secretase Subunit; SASH1: SAM And SH3 Domain Containing 1.

Here, we present a case of DSH in a young boy who recently immigrated to Canada. This report aims to enhance awareness of the clinical presentation of this rare pigmentary genodermatosis and improve diagnostic accuracy of IRPDs in regions where such conditions are seldom encountered.

## Case report

**Figure 1.**
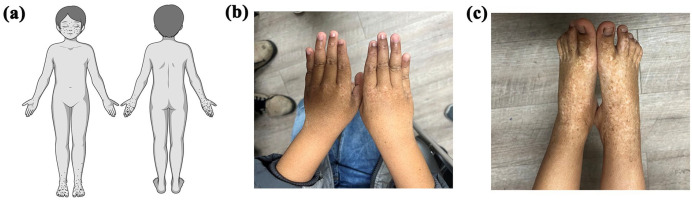
Presentation of DSH in our patient. (a) Visual representation of the typical pattern of pigmentary macules in patients with DSH. (b, c) Mottled hyperpigmented and hypopigmented macules on the dorsal surface of our patient’s hands and feet bilaterally.

A 6-year-old boy of South Asian descent presented to a dermatology clinic in Regina, Saskatchewan for evaluation of numerous asymptomatic pigmented and depigmented macules on his face, hands, and feet (Figure 1). The pigmentary changes began at 16 months of age with involvement of the dorsal hands and feet. Lesions were non-pruritic and non-painful. There was no preceding illness, medication exposure, or identifiable environmental trigger. By age 4, hyperpigmented macules started to develop on the face, and the distribution of lesions progressed proximally along the limbs. The parents expressed concern regarding further spread. Additional cutaneous features of the child include hyperpigmentation over the extensor surfaces of the knees, attributed to chronic mechanical friction from crawling on his knees during play.

Three months prior to our visit, the child immigrated from India to Canada. His past dermatologic history included eczema, which was managed through topical corticosteroids and over-the-counter moisturizers. The patient was of normal stature and behavior for his age. There were no reported abnormalities involving neurologic, cardiovascular, respiratory, or gastrointestinal systems.

The parents had tried to treat his pigmented lesions with topical corticosteroids and over-the-counter lightening creams with no resolution or change. Notably, neither of the child’s biological parents exhibited pigmentary changes or dermatologic conditions, suggesting a de novo presentation of this genodermatosis. To clarify the diagnosis, the patient’s family provided the results of a skin biopsy performed on the left lateral foot in India. Histopathological analysis revealed hyperkeratosis of the granular layer, mild acanthosis with blunted rete ridges, and an intact basal layer, which appeared hypopigmented. There was no significant inflammatory infiltrate observed. Whole-exome sequencing conducted in India identified a heterozygous missense variant in exon 2 of the ADAR gene, resulting in a single-nucleotide substitution of asparagine for lysine at codon 502 (Table 2).

**Table 2.** Result of genetic testing showing a single nucleotide variant identified suggestive of a dyschromatosis symmetrica hereditaria diagnosis for our patient.

Gene	Location	Variant	Zygosity	Disease	Inheritance
ADAR	Exon 2	p.Lys502Asn	Heterozygous	Dyschromatosis symmetrica hereditaria	AD

Such a genetic variant has not been previously reported for a patient with DSH in the literature, suggesting our patient has a novel variant. Correlation with clinical features highly supports the diagnosis of DSH.

AD: autosomal dominant; ADAR: adenosine deaminase acting on RNA.

Based on the patient’s clinical presentation, histopathological findings, and genetic testing, we report a case of DSH in an otherwise healthy 6-year-old boy who recently immigrated to Canada from India. Treatment options for DSH remain limited, and the condition is typically not associated with significant systemic involvement. The family was reassured regarding the benign course of the disorder and advised to seek medical attention should any new systemic or developmental concerns emerge.

## Discussion

Although these presentations may be uncommon in the Canadian population, ongoing immigration from regions such as Asia underscores the importance of recognizing IRPDs, like DSH. Awareness is crucial given that some of these conditions may be associated with systemic manifestations, requiring timely diagnosis and management. DSH is extremely rare, as supported by a PubMed search of “DSH” resulting in a total of 177 papers from the last 75 years. Health professionals may have difficulty diagnosing and addressing these patients’ concerns if they are unaware of such genodermatoses.

Interestingly, DSH is known to be autosomal dominant and has a high penetrance of being passed on within families. However, our patient was the first in his family lineage to have patterned pigmentary changes of his skin, which may suggest a sporadic mutation leading to his clinical presentation. This has been previously reported in the literature as a review of 185 DSH cases in Japan and other counties showed 71 cases without family histories of DSH.^3^ Therefore, the absence of family history should not dismiss a DSH diagnosis. In our patient’s case, dermatologic presentation, skin biopsy, genetic testing, and the absence of additional medical conditions strongly supported his diagnosis. Consistent with prior case reports, his biopsy revealed loss of rete ridges and basal layer hypopigmentation, which are features characteristic of DSH.^15^

The frequency of DSH is unknown and likely underreported, as these patients have pigmentary lesions without serious health complications. Currently, DSH is thought to be a disorder limited to the skin, but few cases have reported neurological symptoms associated with DSH, such as developmental regression and dystonia.^16^ Other associations include hemangioma,^17^ congenital heart disease,^18^ and psoriasis.^19^ Our patient at 6 years of age did not exhibit any correlated conditions.

No therapeutics have been established to be effective for DSH in the literature. One study from China described the use of fractional carbon dioxide laser treatment for DSH; however, further research is needed to confirm its safety and efficacy.^10^ Other treatment options, including topical corticosteroids, pimecrolimus, calcipotriol, and psoralen plus UVA, have been attempted but did not result in the resolution of the condition. Our patient’s lack of response to topical corticosteroids further corroborates this. In conclusion, we highlight the presentation of a young boy of Asian descent with DSH presenting in Canada.
